
JOURNAL INFORMATION
==============================
NLM Title Abbreviation: Behav Neurol
Journal ID: Behav Neurol
NLM Journal ID: 8914585
Journal ID: BN

Behavioural Neurology

ISSN: 0953-4180
EISSN: 1875-8584
Publisher: Wiley

ARTICLE INFORMATION
==============================
PMCID: PMC5434385
PMID: 21422554
DOI: 10.3233/BEN-2010-0294
Article ID: 989286
Article version: 1
Subjects: Research Article

Right Visual Field Attentional Bias in Reading in Children and Adults

Riva Miléna
Siéroff Eric *
Laboratoire de Psychologie et Neuropsychologie Cognitives (CNRS FRE 3292) Institut de Psychologie Université Paris Descartes Boulogne-Billancourt France
*Eric Siéroff: eric.sieroff@parisdescartes.fr
Print publication date: 2010
Electronic publication date: 2011 Mar 7
Volume: 23
Issue: 4
First page: 199
Last page: 201
Received 2011 Jan 31; Accepted 2011 Jan 31
Copyright: Copyright © 2010 Hindawi Publishing Corporation and the authors.
Copyright year: 2010
License: This is an open access article distributed under the Creative Commons Attribution License, which permits unrestricted use, distribution, and reproduction in any medium, provided the original work is properly cited.
License URL: https://creativecommons.org/licenses/by/3.0/

==============================
Behavioural Neurology 23 (2010) 199–201
DOI 10.3233/BEN-2010-0294
IOS Press

### Right visual field attentional bias in reading in children and adults

Miléna Riva and Eric Siéroff*
*Laboratoire de Psychologie et Neuropsychologie Cognitives* (*CNRS FRE 3292*), *Institut de Psychologie, Université Paris Descartes, Boulogne-Billancourt, France*

### 1. Introduction

The identification of parafoveal words is better in the right visual field (RVF) than in the left visual field (LVF) [4]. In experiments using the identification of parafoveal words, attention is not necessarily distributed symmetrically in the state of expectancy prior to the presentation of each stimulus. The RVF superiority may be, at least partially, explained by an attentional bias in favour of the RVF [3], because of the activation of the left hemisphere when subjects are engaged in a linguistic task and because of the reading direction. Although several studies argue in favor of an early development of the RVF superiority for words in children [5], little is known on the development of this attentional bias.

The goal of our study was to investigate the attentional bias in reading parafoveal words in school-age children and adults, using an identification task. Words were presented alone or with a distractor in the opposite visual field. The distractor was simultaneous with the word or was presented immediately before the word. Asimultaneous distractor may compete with the word for the allocation of attention [6,7]. A distractor appearing before the word may capture attention [2]. According to the attentional bias hypothesis we predicted a larger effect of the simultaneous distractor in the LVF, because words in the RVF benefit from more attentional resources. Also, we predicted a similar effect of a distractor preceding the word in both visual fields, or maybe a stronger effect in the RVF, because the RVF bias is interrupted by the capture of attention.

### 2. Methods

18 children of 8 y.o. (third Grade), 18 children of 10 y.o (fifth Grade), and 24 adults (mean age: 21 y.o.) participated to the experiment. 180 4-letter words were used, covering a visual angle of 1.5° and an eccentricity of 1.4° between the fixation item and the nearest extremity of the word. The experiment was run on a Macintosh PowerBook, using Psychlab 2.3 [1]. Each trial began with the presentation of a fixation item for 500 ms in the center of the screen, immediately followed by the word in the LVF or RVF, presented alone or with a distractor (4 xs) in the opposite visual field. The distractor might be presented simultaneously with the word or preceding it (100 ms). The duration of the words was determined in a pre-experiment in order to obtain approximately the same level of correct responses (between 50% and 60%) from all participants. A patterned mask immediately followed words. Participants were required to fixate the fixation item, and to identify and name the words, or spell the letters if the word couldn't be identified. They were specifically instructed to ignore the distractor and not report it. The experimenter, who was positioned in front of the child, monitored eye movements.

*Corresponding author: Pr. Eric Siéroff, Laboratoire de Psychologie et Neuropsychologie Cognitives (CNRS FRE 3292), Institut de Psychologie, Université Paris Descartes, 71, avenue Edouard Vaillant, 92100 Boulogne-Billancourt, France. E-mail: eric.sieroff@parisdescartes.fr.

ISSN 0953-4180/10/$27.50 © 2010 – IOS Press and the authors. All rights reserved

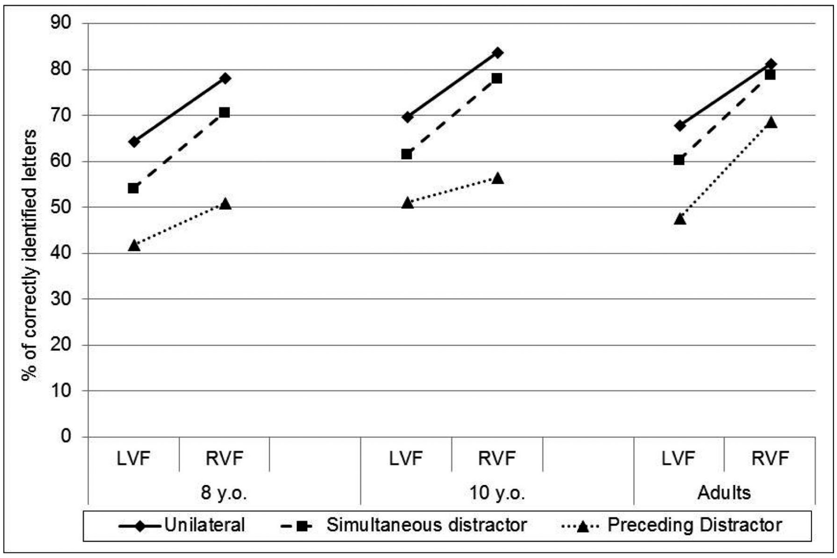

Fig. 1. Percentage of correct letters according to age, visual field and type of presentation.

### 3. Results

Mean presentation times for the words were 141ms in 8 year-olds, 114ms in 10 year-olds and 84ms in adults.

An ANOVA was computed on the percentage of correctly identified letters with Age (8 y.o., 10 y.o., adults) as between-subject factor, and Visual field (LVF, RVF), Type of presentation (single unilateral, unilateral with simultaneous distractor, unilateral with preceding distractor) as within-subject factors.

The significant Age x Type of presentation interaction, $F(4,114) = 3.34$; $p < 0.05$, showed that a simultaneous distractor affected word identification more in children (mean difference between the single and the simultaneous distractor conditions = 7.7%) than in adults (5.8%). Also the preceding distractor affected word identification more in children (mean difference between the simultaneous distractor and the preceding distractor = 16.1%) than in adults (7.9%).

The Type of presentation x Visual field interaction was significant, $F(2,114) = 5.85$; $p < 0.001$, as well as the Age x Type of presentation x Visual field interaction, $F(4,114) = 5.96$; $p < 0.001$ (see Fig. 1). The RVF superiority was equivalent in children (difference between RVF and LVF = 13.8%) and adults (13.4%) in the single unilateral presentation. As predicted, the effect of the simultaneous distractor was larger in the LVF (8.5%) than in the RVF (5.1%), and the asymmetry between visual fields in the unilateral with distractor presentation was equivalent in children (16.4%) and in adults (18.5%). The effect of the distractor preceding the word was larger in the RVF (20.7%) than in the LVF (11.5%) in children, but equivalent in both visual fields in adults (12.9% and 10.4%).

### 4. Discussion

Results showed that word identification was globally affected by the presence of a distractor, and this effect was larger in children than in adults suggesting that reading is more attention demanding in children. Also, word identification was worse with a distractor preceding the word than with a simultaneous distractor, and this effect was larger with RVF than LVF words in children. This result suggests that a RVF attentional bias occurs in the preparatory phase before word presentation, and this bias is reduced when attention is captured by the distractor in the LVF before the presentation of the word. In adults, no asymmetry of the distractor effect was found, as if they inhibit the capture of attention. In conclusion, this study is in favour of a RVF attentional bias in reading, occurring in children as young as 8 y.o.

### References

[1] D. Bub and T. Gum, *PsychLab software*. McGill University, Neurolinguistic Department, Montreal, Canada, 1988.

[2] H.E. Egeth and S. Yantis, Visual attention: Control, representation, and time course, *Annual Review of Psychology* **48** (1997), 269–297.

[3] K.M. Kinsbourne, The cerebral basis of lateral asymmetries in attention, *Acta Psychologica* **33** (1970), 193–201.

[4] M. Mishkin and D.G. Forgays, Word recognition as a function of retinal locus, *Journal of Experimental Psychology* **43** (1952), 43–48.

[5] M.E. Olson, Laterality differences in tachistoscopic word recognition in normal and delayed readers in elementary school, *Neuropsychologia* **11** (1973), 343–350.

[6] E. Siéroff and M. Urbanski, Conditions of visual verbal extinction: Does the ipsilesional stimulus have to be identified? *Brain and Cognition* **48** (2002), 563–569.

[7] A. Treismanand and G. Gelade, A feature-integration theory of attention, *Cognitive Psychology* **12** (1980), 97–136.
